# Efficient and Fast Removal of Oils from Water Surfaces via Highly Oleophilic Polyurethane Composites

**DOI:** 10.3390/toxics9080186

**Published:** 2021-08-05

**Authors:** Antonio De Nino, Fabrizio Olivito, Vincenzo Algieri, Paola Costanzo, Antonio Jiritano, Matteo Antonio Tallarida, Loredana Maiuolo

**Affiliations:** Department of Chemistry and Chemical Technologies, University of Calabria, 87036 Rende (CS), Italy; vincenzo.algieri@unical.it (V.A.); paola.costanzo@unical.it (P.C.); antonio.jiritano@unical.it (A.J.); matteoa.tallarida@unical.it (M.A.T.)

**Keywords:** polyurethane, composite, adsorption capacity, oleophilicity, regeneration

## Abstract

In this study we evaluated the oil adsorption capacity of an aliphatic polyurethane foam (PU **1**) and two of its composites, produced through surface coating using microparticles of silica (PU-Si **2**) and activated carbon (PU-ac **3**). The oil adsorption capacity in diesel was improved up to 36% using the composite with silica and up to 50% using the composite with activated carbon with respect to the initial PU **1**. Excellent performances were retained in gasoline and motor oil. The adsorption was complete after a few seconds. The process follows a monolayer adsorption fitted by the Langmuir isotherm, with a maximum adsorption capacity of 29.50 g/g of diesel for the composite with activated carbon (PU-ac **3**). These materials were proved to be highly oleophilic for oil removal from fresh water and sea water samples. Regeneration and reuse can be repeated up to 50 times by centrifugation, without a significant loss in adsorption capacity.

## 1. Introduction

Oil spillage is one of most common sources of contamination on our planet because, every year, tons of crude and refined materials are transported across the land and sea [1,2,3]. Ground water and sea water represent the easiest targets of contamination [4,5]. Due to the low solubility and biodegradability of oils, the damage to the aqueous ecosystem is permanent until the pollutants are removed [6,7]. The negative effects are not only traceable to aquatic species, but also to the other types of living organisms that use water as a source of life, such as plants and animals [8,9,10]. Fuels, in the form of light hydrocarbons, for example, tars, grease, and diesel oil, or as heavy hydrocarbons, such as kerosene, gasoline, and jet fuel, together with fats, lubricants, and motor oil are increasingly in demand by the market [11,12]. There are many chemical, physical, and biological methods to remove oils from contaminated water [13], including oil dispersant [14], in situ burning [15], filters [16], reverse osmosis [17], gravity separation [18], electrocoagulation [19] and electroflotation [20]. Sorption is probably one of the most used methods because oil sorbents fall into two very broad categories: synthetic, such as polyanilines and polypyrrole-silica polymer composites [21,22], and natural adsorbents, such as bio-based aerogels and natural adsorbents based on sawdust [23,24]. The latter can be further divided into organic and inorganic [25,26]. Synthesis of composite materials employing both synthetic and natural substances is a developing technique and the amount of published research is fast growing [27,28,29]. This methodology allows to exploit both the potential of synthetic materials and the cost-effectiveness of natural ones with the aim of improving chemical-physical characteristics [30,31,32,33]. Polyurethane (PU) foams represent one example of synthetic materials that are widely used for the removal of oils from water, because these polymers possess a large specific area, low density and can be ultralight [34,35]. They are commonly synthesized by the reaction between a polyol and a polyisocyanate, catalyzed in different manners [36,37,38,39]. In our recent work, we developed a green procedure for the synthesis of a series of aliphatic polyurethane foams using a catalyzed two-step approach consisting of prepolymer synthesis and further chain extension [40]. The catalysts used were eco-friendly and the most benign one was sodium chloride. The obtained prepolymers were easily extended without ulterior catalyst addition with short alcohols, such as 1,2-ethylene glycol, to obtain the final polyurethane foams. The replacement of aromatic isocyanates with aliphatic ones, together with the non-toxic catalyst, make these materials more environmentally friendly [41,42]. Polyurethane foams, with their wide porous structures, are optimal materials to incorporate into other substances; for example, to produce composites with enhanced properties [43,44]. Natural compounds, such as silica and activated carbon, possess valuable sorption characteristics to remove many pollutants from wastewater samples [45,46]. There are many recent works that describe the use of composite materials made using a combination of polyurethanes and natural compounds [47,48]. In this work, we propose the facile production of one polyurethane foam and two polyurethane composites derived from it by surface coating, employing microparticles of silica and activated carbon. The main purpose of this project is the preparation of porous materials with a low density and high absorptive capacity. In particular, the lipophilic nature of the obtained composite materials confers a high affinity for petroleum derivatives to remediate polluted water, even on a large scale.

During this work, we carried out a comparison of the three types of materials and for all adsorption followed a monolayer mechanism, typical of a Langmuir isotherm. This model has been commonly used to define the phenomenon of adsorption and is based on the statement that the adsorption process occurs at particular homogeneous sites on the surface of an adsorbent with a monolayer filling of non-interacting molecules. Recent applications reported an effective sorption study on a polyurethane foam functionalized with salicylate as adsorbent for chlorpyrifos extraction, rationalizing the process by the Langmuir isotherm [49]. A similar work but targeting different pollutants was conducted using a polyurethane/polysaccharide material [50].

The products tested can be regenerated up to 50 times using simple centrifugation, and the two composites showed higher performances with respect to the initial PU for the adsorption of diesel, gasoline, and motor oil, both from fresh water and sea water samples. The products were characterized by FT-IR and the morphology was analyzed by SEM.

## 2. Materials and Methods

### 2.1. Chemicals

Polyethylene glycol, PEG 400, was purchased from Thermo Fisher Scientific, Rodano (MI), Italy. Molecular formula: H(OCH_2_CH_2_)_n_OH, *n* = 8–9 (average), at a 99% purity grade. Isophorone diisocyanate (IPDI) was purchased from EVONIK INDUSTRIES, Essen, Germany at a 95% purity grade. Anhydrous sodium chloride was purchased from Sigma Aldrich-Merk Life Science S.r.l., Milano, Italia and was of analytical grade. Ethylene glycol was purchased from Carlo Erba, Cornaredo Milano, Italy at a purity grade >99%. Silica was purchased from Merck Life Science S.r.l., Milano, Italy at a 90% purity grade and particle size <45 µm. Activated carbon was purchased from Sigma Aldrich-Merk Life Science S.r.l., Milano, Italy at a high purity level and particle size <100 µm. Diesel, unleaded gasoline, and certified waste motor oil were purchased from Sigma Aldrich-Merk Life Science S.r.l., Milano, Italia.

### 2.2. Synthesis of Polyurethane ***1***

#### 2.2.1. Prepolymer Synthesis

The prepolymer was synthesized using isophorone diisocyanate (IPDI) in a molar excess of 2.5:1 with respect to PEG 400. Water was used as a blowing agent in a weight percentage of 5.6% with respect to PEG 400. The reaction was monitored using FT-IR spectroscopy with respect to the isocyanate signal. The produced prepolymer was defined as stable in accordance to the standard titration method, ASTM D 2572-97, using di-*n*-butylamine [40]. The reagents were added in the following order to a plastic container: PEG 400, distilled water, and salt. The mixture was stirred using a mechanical apparatus. Diisocyanate was added and the mixture was vigorously stirred. The mixture was warmed up to 70 °C for one hour until diisocyanate consumption occurred. The prepolymer was obtained in the form of a colourless gel.

#### 2.2.2. Chain Extension

Ethylene glycol, in a quantity of 30% by weight with respect to the prepolymer, was added to the same reaction container. This ratio was discovered by FT-IR investigations as the equivalent ratio for complete isocyanate group functionalization. The mixture was vigorously stirred and poured in a steel mold under pressure. The mixture was warmed up to 70 °C for one hour. After that, the produced polyurethane foam (**1**) was left to cure at room temperature for two weeks. The polyurethane signal disappearing was determined by FT-IR characterization and proved the completion of the reaction.

### 2.3. Preparation of Composites ***2*** and ***3***

The produced polyurethane foam, **1**, was cut into cubes with a dimension of approximately 1 cm × 1 cm. The polyurethane cube was mixed in diethyl ether (approximately 10 mL) and added to a one weight equivalent with respect to the used polyurethane of microparticles of silica or activated carbon We chose this quantity because preliminary tests demonstrated an incomplete surface coating by using less than one equivalent of microparticles, while employing larger amounts of the composite coating was the same as that obtained using one equivalent.

The mixture was stirred for one hour and then the obtained composites (**2** with silica and **3** with activated carbon) were washed with fresh diethyl ether (30 mL) to remove the microparticles that were not adsorbed by the surface. The composite was dried in an oven at 50 °C for one day. Stability tests were carried out based on the possible weight loss of the composites. The two composites did not show any weight loss after 50 regeneration cycles and after 50 washes with common organic solvents. The composites were also proved to be stable at temperatures higher than room temperature.

### 2.4. Adsorption Capacity Test in Oil Phase

The adsorption capacity in the oil phase was determined in accordance to the ASTM F726-06 standard method [51]. Polyurethane foam was cut into known dimensions and weighed. The sorbent was put in 50 mL of oil phase (diesel, gasoline, motor oil) for a defined time. Subsequently, the material was removed and weighed. The oil adsorption capacity, S, was calculated using Equation (1):

(1) $$S\;\left(g/g\right)=\frac{S_{t}-{\;S}_{0}}{S_{0}}$$

where S_t_ is the weight of the material after adsorption and S_0_ is the initial weight of the material.

### 2.5. Adsorption Capacity Test in Oil/Fresh Water System

The adsorption capacity in oil/fresh water system was determined as reported elsewhere [52]. The sorbent in the form of cubes was put into the oil/water system under mixing, using different concentrations. After a defined time, the material was removed and placed in a centrifuge using a centrifugal concentrator tube to collect the oil (Vivaspin^TM^ 6 centrifugal concentrator tubes were purchased from Fischer Scientific). The adsorbed materials were centrifuged at 500 rpm with a rotor radius of 11 cm for 1 min until the oil was separated on the bottom. The regeneration could be repeated up to 50 times. The collected biphasic system consisting of oil with a small percentage of water was subjected to liquid/liquid extraction, using three portions of petroleum ether to extract the organic phase. The organic phases were collected and dried over anhydrous sodium sulphate. The mixture was filtered using a sintered glass funnel in a one-neck round bottom flask and the solvent was removed under reduced pressure. The oil adsorption capacity was calculated using Equation (2):

(2) $$S\;\left(g/g\right)=\frac{M_{t}-{\;M}_{0}}{S_{0}}$$

where M_0_ is the weight of the reaction flask, M_t_ is the weight of the reaction flask plus the collected oil after the process and S_0_ is the initial weight of the sorbent.

The oil adsorption percentage Pa (%) reported in Equation (3) was plotted against the initial concentration C (g/L).

(3) $$\mathrm{Pa}\;\left(\%\right)=\frac{O_{a}\;}{O_{t}}\times 100$$

where O_a_ is the quantity of the adsorbed oil after the process and O_*t*_ is the initial weight of the oil in water.

### 2.6. Adsorption Capacity Test in Oil/Sea Water System

The adsorption capacity was calculated in the same manner of the previously described method for oil/fresh water system. The effect of a solution with different ionic strength was evaluated on the adsorption. Solutions of different concentrations were prepared adding the oil on the surface of synthetic seawater prepared by the ASTM D1141-98 [51].

### 2.7. Adsorption Isotherms

The Langmuir model is relative to a monolayer adsorption onto a homogeneous surface in which the reactive groups of the sorbent are homogeneously distributed on the structure. This model is described by Equation (4):

(4) $$\mathrm{Langmuir}\;\mathrm{isotherm}:\;\frac{C_{e}}{q_{e}}=\frac{1}{k_{L}q_{m}}+\frac{1}{q_{m}}C_{e}$$

where the Langmuir constants q_m_ and k_L_ represent the maximum adsorption capacity of the adsorbent and the constant energy related to the heat of adsorption, while C_e_ (mg/L) is the concentration of adsorbate in the liquid phase at equilibrium and q_e_ (mg/g) is the amount of adsorbate adsorbed on the solid phase at equilibrium.

One of the essential characteristics of the Langmuir isotherm can be expressed by a dimensionless constant separation factor (R_L_), defined in Equation (5):

(5) $$R_{L}=\frac{1}{1+k_{L}C_{0}}$$

the value of R_L_ indicates the type of the isotherm; which is unfavorable (R_L_ > 1), linear (R_L_ = 1), favorable (0 < R_L_ < 1), or irreversible (R_L_ = 0).

The Freundlich isotherm can be related to multilayer adsorption or adsorption on heterogeneous surface. The mathematical formula of the Freundlich isotherm is reported in Equation (6):

(6) $$\mathrm{Freundlich}\;\mathrm{isotherm}:{\mathrm{lnq}}_{e}={\mathrm{lnk}}_{F}+\frac{1}{n}{\mathrm{lnC}}_{e}\;$$

where k_F_ (mg/g) (L/mg)^1/*n*^ indicates the adsorption capacity, and *n* reflects the intensity of adsorption according to the Freundlich theory, q_e_ is the equilibrium weight of adsorbate per unit weight of adsorbent (g/g), C_e_ is the concentration of adsorbate in solution at equilibrium after adsorption (g/L).

### 2.8. FT-IR and SEM Characterization

FT-IR spectra (KBr pellets) were recorded with a PerkinElmer Spectrum One FT-IR spectrometer in a frequency range between 450 and 4000 cm^−1^. Morphological studies of PU-Si **2** and PU-ac **3** were carried out using a Phenom ProX (Thermo Fisher Scientific) scanning electron microscope (SEM), operating under vacuum conditions of 8 × 10^−6^ Torr at an accelerating voltage of 15 kV.

## 3. Results and Discussion

**Figure 1:**
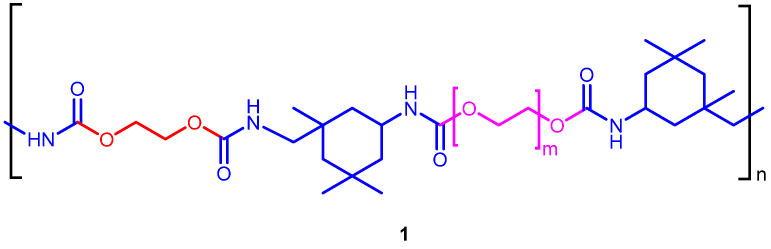
Structure of polyurethane **1**.

The starting polyurethane PU **1** was prepared through a simple two-step procedure (prepolymer synthesis and elongation step) using PEG 400, isophorone diisocyanate (IPDI) and 1,2-ethylene glycol as a chain extender [40]. The catalyst used was sodium chloride while water was the blowing agent. In Figure 1 the chemical structure of resulting polyurethane **1** is represented.

**Figure 2:**
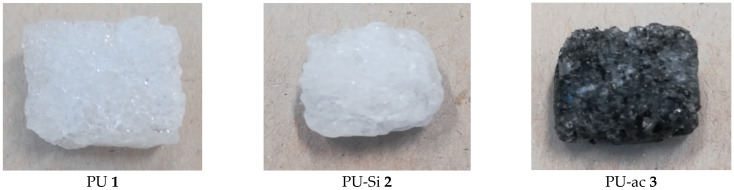
Photographs of polyurethane **1** and composite materials **2** and **3**.

Then, the composites PU-Si **2** and PU-ac **3** were obtained in one hour, by simply mixing the PU **1** with a suspension of the microparticles of silica or activated carbon in diethyl ether. In Figure 2, photographs of PU **1**, PU-Si **2** and PU-ac **3** are reported.

### 3.1. Characterization of the Materials Used in this Study

#### 3.1.1. FT-IR Spectra

FT-IR spectra of the starting polyurethane **1** is the same reported elsewhere [40]. The spectra of the composites PU-Si **2** and PU-ac **3** showed almost the same peaks and there is not an appreciable difference among these spectra (a comparison of FT-IR spectra is reported in Supplementary Figure S2). The percentage of microparticles of silica or activated carbon after the coating of the polyurethane surface is around 5% in weight, a value that does not allow distinct and significant peaks other than polyurethane signals. The only variation is relative to the broadness and the intensity of the peaks. This is in line with the characterization of the common polyurethane composites reported in literature [53,54]. In the case of the two newly synthesized composites (**2** and **3**), the characteristic N–H peak at 3357 cm^−1^ and C–O peak at 1126 cm^−1^ became broader and weaker with respect to PU, this is due to various interactions between the urethane chains and the microparticles, among which, the most important are hydrogen bonds. This is the qualitative proof of the presence of silica or activated carbon microparticles on the surface of the polyurethane.

#### 3.1.2. SEM Analysis

**Figure 3:**
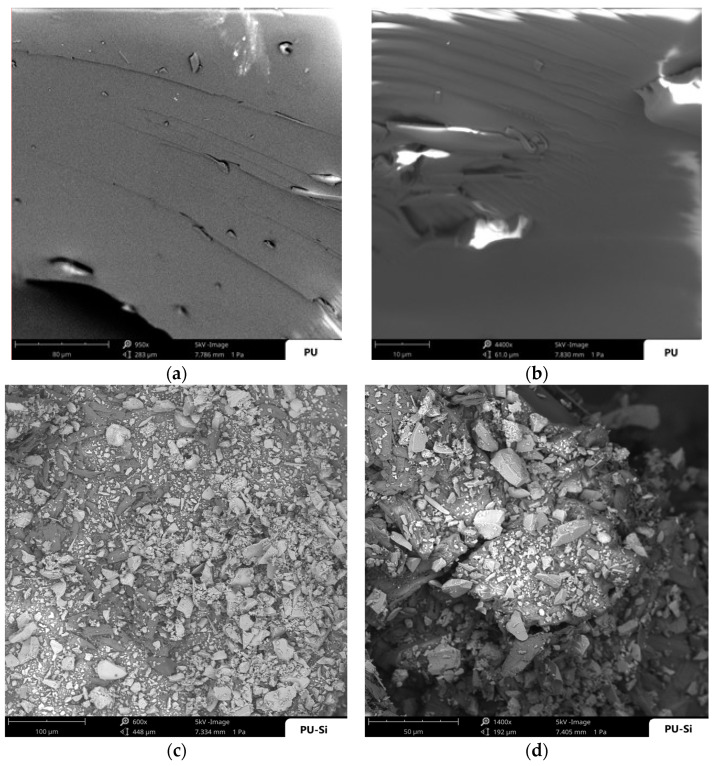

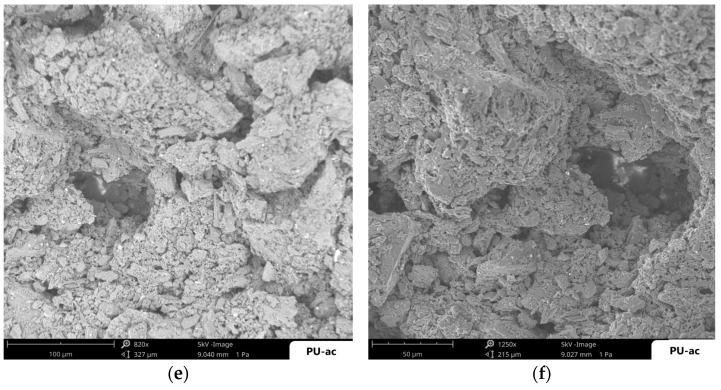
(**a**,**b**) SEM images of the starting polyurethane **1** at different magnifications; (**c**,**d**) SEM images of the polyurethane composite PU-Si **2** at different magnifications. (**e**,**f**) SEM images of the polyurethane composite PU-ac **3** at different magnifications.

The morphological analysis was performed using SEM and revealed the complete surface coating of the polyurethane composites obtained with silica (**2**) and activated carbon (**3**), Figure 3. Images (a) and (b) show the starting polyurethane **1**, without microparticle coatings or other impurities with a homogenous and compact surface. Images (c) and (d) show the polyurethane composite PU-Si **2** and prove the presence of silica microparticles on the polymer surface; in particular, it is evident that these particles are distributed in a double layer, in which the bottom layer is more uniform. Activated carbon microparticles also provide a complete coating of the polyurethane surface, how it is shown in images (e) and (f) for polyurethane composite PU-ac **3**, but, in this case, the homogenous agglomerate appeared as single layer.

### 3.2. Regeneration and Reuse in Diesel

**Figure 4:**
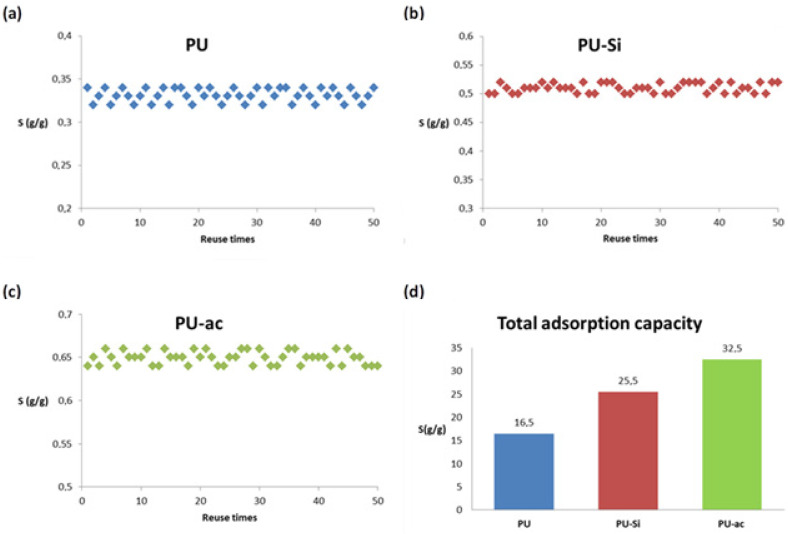
(**a**) Reuse of polyurethane **PU 1** in diesel after 50 regeneration cycles. (**b**) Reuse of polyurethane composite **PU-Si 2** in diesel after 50 regeneration cycles. (**c**) Reuse of polyurethane composite **PU-ac 3** in diesel after 50 regeneration cycles. (**d**) Total adsorption capacity for the three types of tested materials.

For simplicity, we decided to evaluate the capacity of regeneration and reuse for the three materials, PU **1**, PU-Si **2** and PU-ac **3**, only in diesel. PU **1** together with the other two types of composites (PU-Si **2** and Pu-ac **3**) were proved to be highly reusable using simple centrifugation for oil desorption. The low density of the three materials together with the large volume of their pores allows a reversible interaction with the adsorbed oil. Centrifugation was proved to be more efficient than other methods, such as solvent extraction or mechanical wringing. The materials can be regenerated and reused up to 50 times without a significant loss in adsorption capacity or any damage to the structure. The adsorption is almost instantaneous and the materials do not precipitate in the oil phase after adsorption, this is important for the recovery of adsorbents from the surface. We report in the following (Figure 4) graphs (a–c) relative to the regeneration of the materials tested, plotting the single sorption capacity S (g/g) with respect to the number of reuse cycles for PU **1** (a), PU-Si **2** (b) and PU-ac **3** (c). In plot (d), we report the values of the total adsorption capacity for the three type of materials. The best performance was obtained for the composite with activated carbon, PU-ac **3**, with a value of 32.5 g/g after 50 cycles.

### 3.3. Adsorption Capacity Using Different Oil Phases

**Figure 5:**
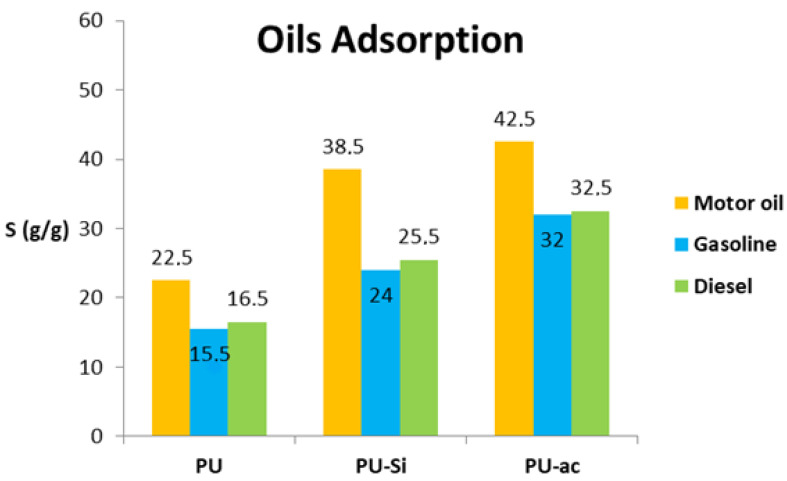
Oil adsorption capacity, S, respect to different types of oils.

We evaluated the total adsorption capacity for the three materials PU **1**, PU-Si **2** and PU-ac **3** in diesel, gasoline, and motor oil. The best performance was obtained for motor oil, which is the denser of the tested oily phases. This behavior is probably due to the fact that a denser phase is easily captured and sequestered inside the polyurethane pores [52]. The microparticle coating of the composite surfaces by silica (PU-Si **2**) and activated carbon (PU-ac **3**) produced an enhanced capacity of adsorption for both with values of 38.5 g/g and 42.5 g/g, respectively. The adsorption is lower in the case of other oil phases, such as gasoline and diesel, but the values are excellent with respect to other common sorbents, which will be discussed at the end of this section. Additionally, for these phases, the values obtained are higher for the composite PU-Si **2** and PU-ac **3** with respect to PU **1**. All three types of tested materials showed optimal performances despite the chemical compositions relative to the heavy hydrocarbons contained in gasoline and the light hydrocarbons contained in diesel. The values are reported in Figure 5.

**Figure 6:**
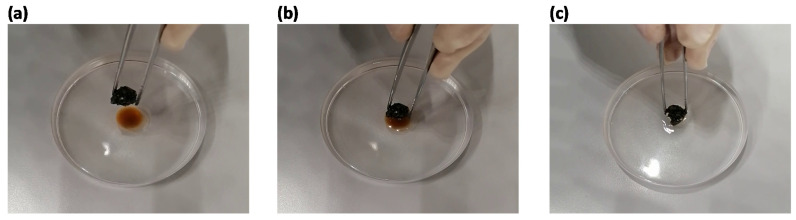
(**a**) Photograph of composite 3 before entering into contact with the surface; (**b**); photograph of composite 3 during contact with the surface. (**c**) Photograph of composite 3 after contact with the surface.

In Figure 6, a photographic sequence is reported depicting the complete removal of motor oil in fresh water by composite PU-ac **3** (Videos S1–S3 are reported in the Supplementary Materials).

As anticipated, in Table 1, we report a comparison between the adsorption capacity of some of the polyurethane composites recently reported in the literature and composites **2–3**, produced and tested in our study. Composite PU-Si **2** furnishes an adsorption capacity higher or comparable with respect to other materials, while composite PU-ac **3** shows higher values with respect to all the materials reported. Almost all the works report composite preparations carried out by chemical functionalization or inclusion in reaction mixture, while this method proposes a simple production through surface coating.

### 3.4. Effect of Initial Concentration in Diesel/Fresh Water System

**Figure 7:**
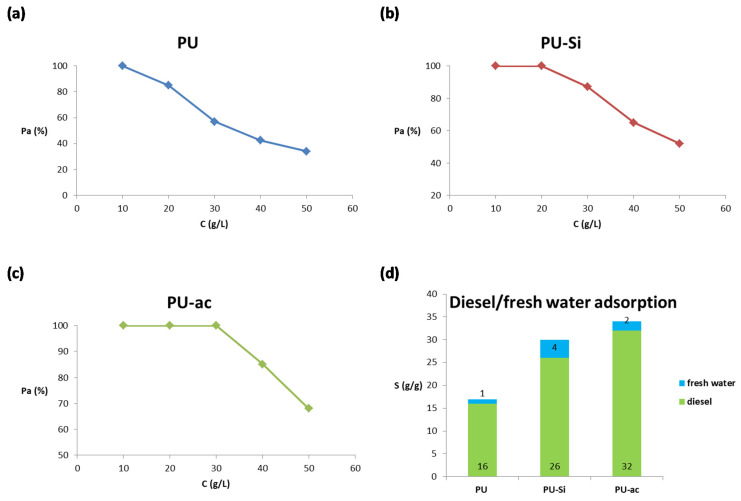
(**a**) Percentage of adsorption for PU 1 at different starting concentrations; (**b**) percentage of adsorption for PU-Si 2 at different starting concentrations; (**c**) percentage of adsorption for PU-ac 3 at different starting concentrations; (**d**) total adsorption capacity for the three types of materials, 1–3, tested with respect to diesel and fresh water.

We investigated the adsorption of materials 1–3 using different concentrations of diesel in fresh water, as reported in the literature [60]. We plotted the oil adsorption percentage, Pa, with respect to five different concentrations, 10, 20, 30, 40, and 50 g/L, and the graphs are reported in Figure 7. From the graph in Figure 7a, it is possible to observe that PU **1** shows an adsorption capacity that is dependent on the concentration. The adsorption capacity is maximum with an oil removal of 100% starting at a concentration of 10 g/L. The adsorption capacity is lower at other higher concentrations, with a value of 34% of percentage of removal starting from a solution of 50 g/L. Composite PU-Si 2, from the graph in Figure 7b, shows an almost complete oil removal at 10 g/L and 20 g/L, while at the other higher concentrations, the percentage of removal became lower with a value of 52% starting from a concentration of 50 g/L. The trend is slightly different for the composite with activated carbon, PU-ac 3, because, from the graph in Figure 7c, it is shown that there was a complete oil adsorption using three different starting concentrations of oil, 10, 20 and 30 g/L. The percentage of removal furnishes a value of 85% starting from a solution of 40 g/L and 68% using a starting solution with an oil concentration of 50 g/L. Probably, the best performance of PU-ac 3 can be related to its greater lipophilicity due to the disorganized graphite form of the activated carbon. In the last graph (Figure 7d) we report the total quantity of diesel and water adsorbed by the three type of materials. From this plot, it is evident that all the materials tested were highly oleophilic with a strong selectivity for the oil phase in oil/fresh water systems.

### 3.5. Adsorption Isotherms in Diesel/Fresh Water System

**Figure 8:**
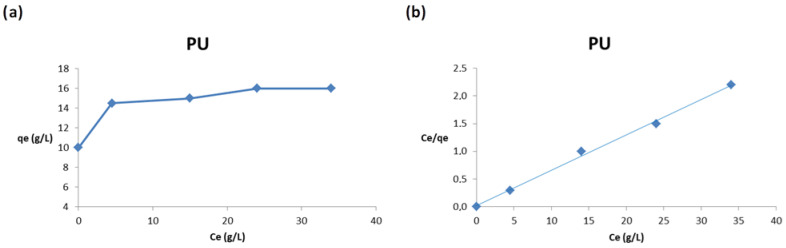
(**a**) Effect of the initial concentration on the adsorption capacity, q, (**b**) Langmuir plot.

We investigated the adsorption capacity of PU **1**, PU-Si **2** and PU-ac **3** with respect to different initial concentrations of oil in fresh water. We report in Figure 8 the plots (a,b) related to PU **1**, in a range of concentrations, from 10 to 50 g/L.

When the concentration of 10 g/L was used, the adsorption was complete because the porous material was able to incorporate almost all the diesel present in the solution. The adsorption capacity, q, increases significantly from 10 to 20 g/L, after that the increase is slow until the plot reaches a characteristic plateau at 40 g/L, due to the saturation of the adsorption sites, as reported in Figure 7a. The obtained values are properly fitted by the Langmuir isotherm, as shown in Figure 7b.

**Figure 9:**
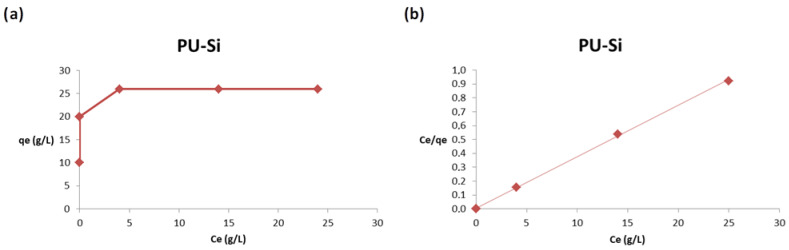
(**a**) Effect of the initial concentration on the adsorption capacity, q, (**b**) Langmuir plot.

We report the same plots (a,b) for composite PU-Si 2 in Figure 9.

For composite PU-Si 2, the adsorption is complete for both the starting concentrations of 10 and 20 g/L. The adsorption capacity increases significantly from 10 to 30 g/L, after that there was a characteristic plateau relative to the saturation of the adsorbent, as illustrated in Figure 8a. Additionally, for this material, the values were properly fitted by the Langmuir isotherm, as shown in Figure 8b.

**Figure 10:**
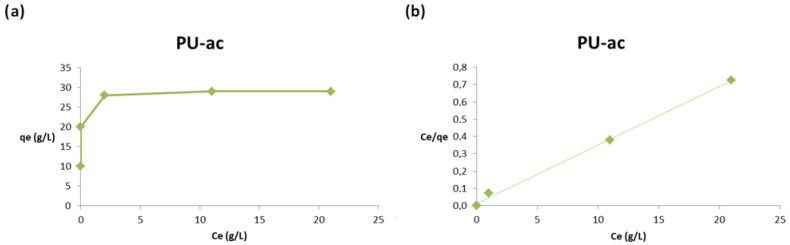
(**a**) Effect of the initial concentration on the adsorption capacity, q, (**b**) Langmuir plot.

Finally, composite PU-ac 3 was investigated and the plots are reported in Figure 10.

The trend is similar to the composite with silica, but the adsorption capacity is higher at all concentrations used, as shown in Figure 10a. The obtained results are also properly fitted using the Langmuir isotherm as reported in Figure 10b.

In Table 2 we report the isotherm parameters for the Langmuir and Freundlich models. The plots relative to the Freundlich model are reported in the Supplementary Figure S1.

The best correlation coefficients (R^2^) were obtained from the Langmuir model, this is evidence that the process follows a monolayer type adsorption for the three type of materials tested, **1**–**3**. The dimensionless constant, R_L_, which is an equilibrium parameter to define if the process is favorable or not, in this case, lies between 0.01 and 0.06 for PU **1**, 0.03 and 0.12 for PU-Si **2** and 0.02 and 0.08 for PU-ac **3**. The values are related to the favorable Langmuir isotherm (single values are reported in Supplementary Table S1). The Freundlich model does not produce a good fit because the process follows an adsorption typical of a finite number of sites distributed on the surface in a homogenous manner. The composites show better adsorption at a concentration range of 10–30 g/L. From the Langmuir model, the obtained maximum adsorption capacity was 29.50 g/g for the composite with activated carbon PU-ac **3** and 26.95 g/g for the composite with silica PU-Si **2**.

### 3.6. Adsorption Capacity in Diesel/Seawater System

**Figure 11:**
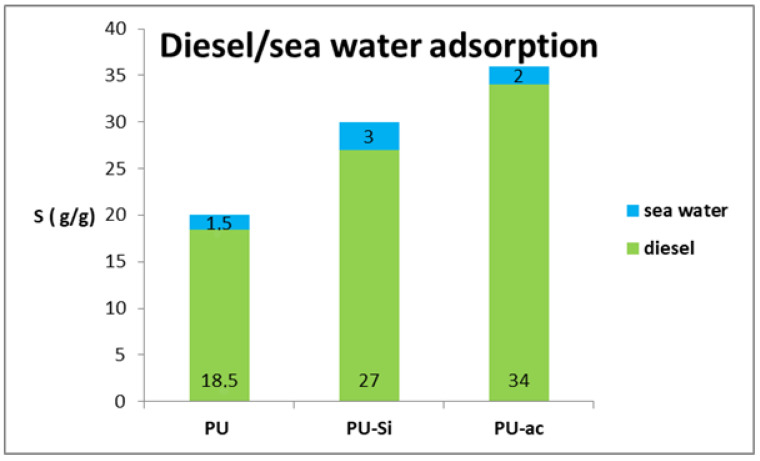
Total adsorption capacity for the three types of materials, **1–3**, tested with respect to diesel and sea water.

We evaluated the adsorption capacity in a system with the presence of different ions in the aqueous solution. It is well known that the presence of many electrolytes enhances the repulsion of a hydrophobic material and water; in contrast, the affinity for the organic phase is strengthened. This phenomenon can surely be related to the salting-out effect [61]. For this reason, the adsorption capacity for the oil phase is slightly higher with respect to a diesel/fresh-water system. The values are reported in Figure 11.

### 3.7. Video Recording

We prove according to Videos S1–S3 in Supplementary Materials the oil selectivity of the three types of materials evaluated in this study, PU **1**, PU-Si **2** and PU-ac **3**. From the videos, the instantaneous and almost complete oil adsorption in a system composed of motor oil and fresh water using a Petri dish is evident.

## 4. Conclusions

In this work, we prepared two polyurethane composites (**2** and **3**) using a simple surface coating method starting from an aliphatic polyurethane **1**. For this purpose, we used two abundant natural products, silica and activated carbon, and we obtained the composites simply after mixing polyurethane **1** in a suspension of their microparticles. We evaluated the adsorption capacity of oil phase in oil/fresh water and in oil/sea water systems. The reported results proved that the performances of composites **2** and **3** are strongly enhanced with respect to the starting polyurethane **1**. Adsorption capacity was evaluated for the three materials, **1–3**, in diesel, gasoline, and motor oil. The obtained results showed that the composite with activated carbon (PU-ac **3**) is the best material and the most promising for future applications. The obtained values are comparable or higher respect to the previous reported studies. The composites are easily regenerated by centrifugation and can be reused up to 50 times without a significant loss with respect to the adsorption capacity.

Therefore, considering the low cost of the starting materials, the simple and efficient production procedure of the materials and their fast regeneration through simple centrifugation, we think that our composites can be widely used in industrial applications on a larger scale.

## Figures and Tables

**Table 1:** A comparison between the materials reported in the literature and composites **1–3** tested in this study.

Composite	Oil	Adsorption Capacity (g/g)	Preparation	Reference
PU-MWCNT	Crude oil	23.0	Chemical functionalization	[55]
PU-nanoclay	Crude oil	21.5	Inclusion in reaction mixture	[56]
PU-palm fiber	Diesel	28.9	Inclusion in reaction mixture	[57]
	Crude oil	19.3		
PU-graphite	Diesel	20.0	Surface coating	[58]
	Motor oil	26.0		
PU-lignin	Crude oil	28.0	Chemical functionalization	[59]
PU-Si **2**	Diesel	25.5	Surface coating	Our study
	Gasoline	24.0		
	Motor oil	38.5		
PU-ac **3**	Diesel	32.5	Surface coating	Our study
	Gasoline	32.0		
	Motor oil	42.5		

**Table 2:** Isotherm parameters for diesel adsorption.

Isotherm	Parameter	PU	PU-Si	PU-ac
Langmuir	K_L_	1.6	0.74	1.13
	q_m_	15.63	26.95	29.50
	R^2^	0.997	0.999	0.998
Freundlich	K_F_	2.83	3.26	3.37
	*n*	8.18	4.91	4.47
	R^2^	0.765	0.522	0.474

## Data Availability

Data are contained within the article and Supplementary Materials.

## Supplementary Materials

The following are available online at https://www.mdpi.com/article/10.3390/toxics9080186/s1. Figure S1: (a) Freundlich plot for PU **1**; (b) Freundlich plot for PU-Si **2**; (c) Freundlich plot for PU-ac **3** [62]. Table S1: R_L_ values at several initial concentrations. Figure S2. FT-IR spectra of **1–3** Video S1: Motor oil adsorption by PU **1**. Video S2: Motor oil adsorption by PU-Si **2**. Video S3: Motor oil adsorption by PU-ac **3**.
