# Supplementary material for: Efficient and Fast Removal of Oils from Water Surfaces via Highly Oleophilic Polyurethane Composites
Source: Toxics. 2021 Aug 5;9(8):186. doi: 10.3390/toxics9080186 (PMC8402441; doi:10.3390/toxics9080186)
Supplement: Supplementary file 1 [file toxics-09-00186-s001.zip › toxics-1311253 - supplem final.pdf]

toxics

Article

Supplementary Material: Efficient and Fast Removal of Oils from Water Surfaces Via Highly Oleophilic Polyurethane Composites

Antonio De Nino *, Fabrizio Olivito *, Vincenzo Algieri, Paola Costanzo, Antonio Jiritano, Matteo Antonio Tallarida and Loredana Maiuolo *

1. Freundlich Isotherms

(a)

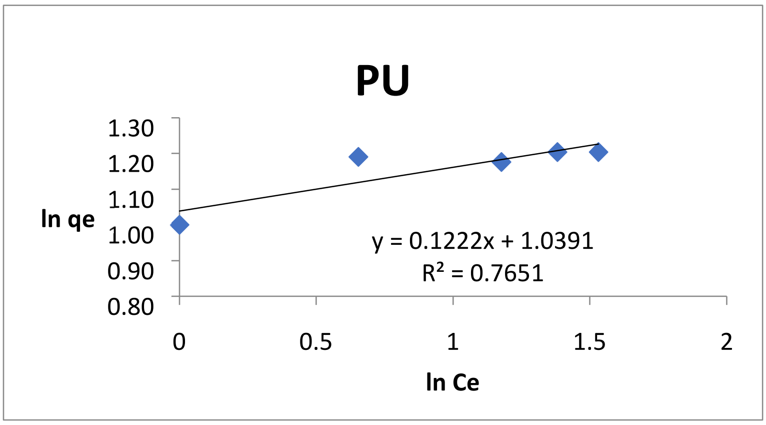

(b)

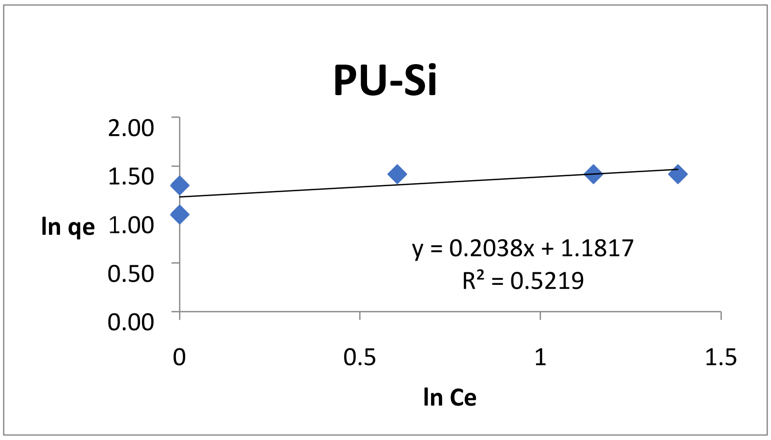

(c)

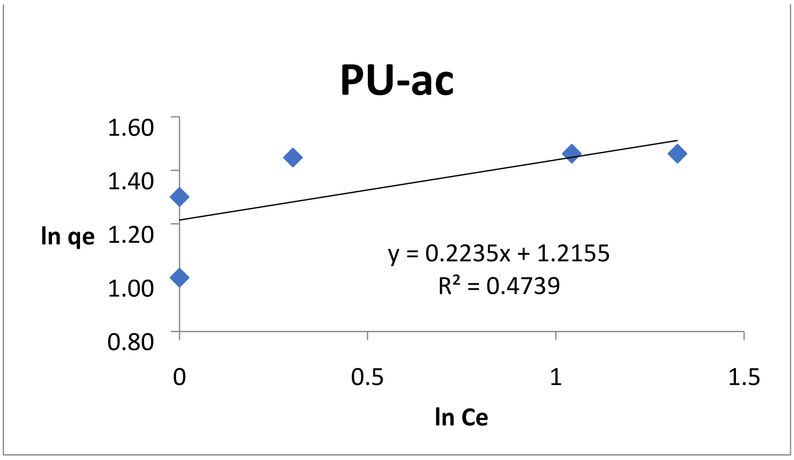

Figure S1. (**a**) Freundlich plot for PU **1**; (**b**) Freundlich plot for PU-Si **2**; (**c**) Freundlich plot for PU-ac **3**.

Freundlich isotherm:

$$lnq_e = lnk_F + \frac{1}{n}lnC_e \quad (1)$$

where C_e (mg/L) is the concentration of adsorbate in the liquid phase at equilibrium and q_e (mg/g) is the amount of adsorbate adsorbed on the solid phase at equilibrium. k_F (mg/g) $(L/mg)^{1/n}$ indicates the adsorption capacity, and n reflects the intensity of adsorption according to the Freundlich theory.

2. Langmuir Dimensionless Constant

One of the essential characteristics of the Langmuir isotherm can be expressed by a dimensionless constant, separation factor, R_L, defined as follows:

$$R_L = \frac{1}{1 + k_L C_0} \quad (2)$$

The value of R_L indicates the type of the isotherm, which is unfavorable ($R_L > 1$), linear ($R_L = 1$), favorable ($0 < R_L < 1$) or irreversible ($R_L = 0$).

In the following Table we report the R_L values for each starting concentration of oil used:

Table S1. R_L values at several initial concentrations.

C_0	R_L PU	R_L PU-Si	R_L PU-ac
10	0.06	0.12	0.08
20	0.03	0.06	0.04
30	0.02	0.04	0.03
40	0.02	0.03	0.02
50	0.01	0.03	0.02

3. FT-IR Spectra

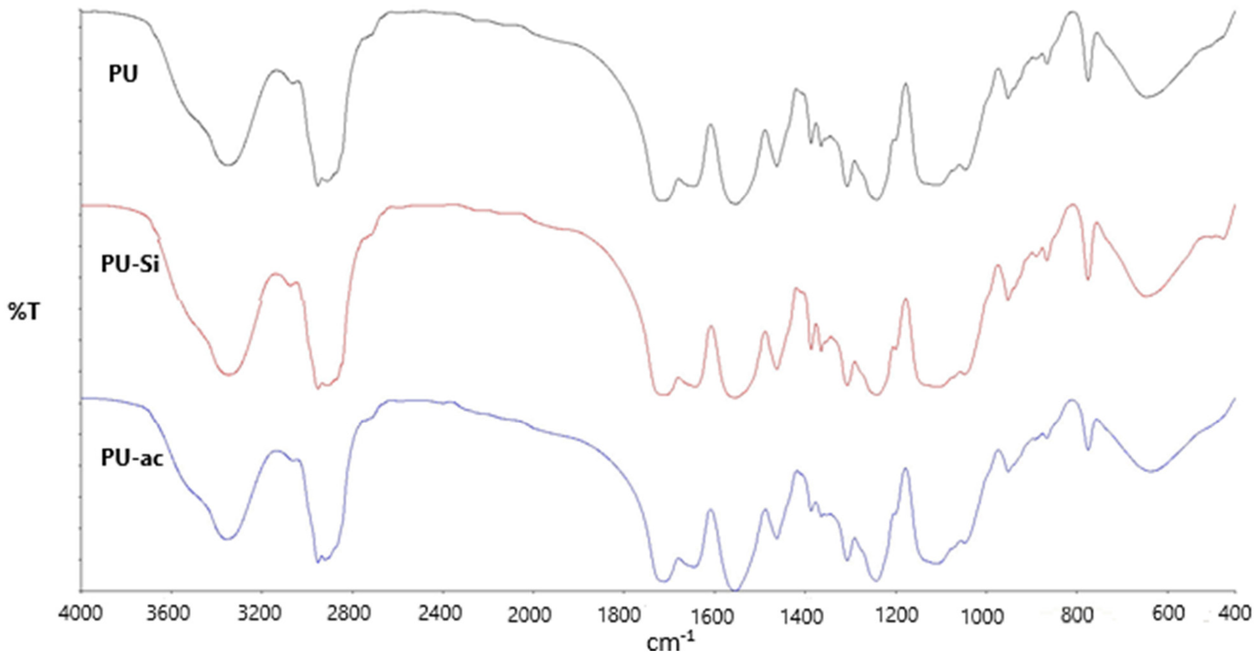

Figure S2. FT-IR spectra of **1**–**3**.

References

1. Kumari, S.; Chauhan, G.S.; Ahn, J.H. Novel cellulose nanowhiskers-based polyurethane foam for rapid and persistent removal of methylene blue from its aqueous solutions. *Chem. Eng. J.* **2016**, *304*, 728–736.
